# Total pancreatoduodenectomy for multiple pancreatic cysts in von Hippel-Lindau disease presenting as obstructive jaundice: A case report

**DOI:** 10.1016/j.ijscr.2023.108481

**Published:** 2023-07-14

**Authors:** Wifanto Saditya Jeo, Khalikul Razi, Andre Setiawan, Natasya, R. Welly Hartono

**Affiliations:** a Division of Digestive Surgery, Faculty of Medicine Universitas Indonesia, Dr. Cipto Mangunkusumo General Hospital, Jakarta, Indonesia; b Department of Digestive Surgery, Siloam Hospital Kebon Jeruk, Jakarta, Indonesia; c Department of Pathology Anatomy, Siloam Hospital Kebon Jeruk, Jakarta, Indonesia

**Keywords:** Pancreatic cyst, Total pancreatoduodenectomy, von Hippel-Lindau disease, Case report

## Abstract

**Introduction:**

Von Hippel-Lindau (VHL) disease can be known as a rare autosomal dominant syndrome that affects some organ systems and is characterized by the growth of both benign and malignant tumors. Diagnosis and management of VHL were needed to have better outcomes.

**Case presentation:**

A 39-year-old male with a history of VHL disease and positive family history presented with jaundice and pruritus. He had a history of craniotomy thrice. Laboratory workup revealed elevated total bilirubin level with conjugated bilirubin predominant. The contrast-enhanced MRI showed dilatation of biliary tree with suspicion of partial obstruction by multiple cysts in the pancreas, with ±0.5–5 cm in diameter. A PET/CT scan showed multiple lesions corresponding to VHL disease. The patient underwent total pancreatoduodenectomy. The histopathology finding was multicystic pancreatic hamartoma with neuroendocrine cell hyperplasia.

**Clinical discussion:**

Multiple pancreatic cysts without prior pancreatic inflammatory episodes should suggest VHL disease and prompt a genetic test, according to clinical presentation. As soon as the diagnosis is made, all potential family members must be screened, and those who are affected must receive genetic counseling and strict follow-up care to treat the disease's potentially fatal CNS and visceral manifestations. Total pancreatoduodenectomy was performed according to jaundice, risk of pancreas malignancy, and the existence of endocrine pancreatic insufficiency.

**Conclusion:**

Total pancreatoduodenectomy could be performed to relieve the symptom severity and avoid the possibility of malignant changes in VHL.

## Highlights

• Von Hippel-Lindau (VHL) disease is a rare autosomal dominant syndrome that affects several organ systems and cause the formation of both benign and malignant tumors
• Multiple pancreatic cysts without prior pancreatic inflammatory episodes should suggest VHL disease
• Suggestion of VHL disease should prompt a genetic test, according to clinical presentation
• The decision to have a total pancreaticoduodenectomy depended on the patient's severe symptomology and to avoid the possibility of malignant changes in VHL

## 1 Introduction

Von Hippel-Lindau (VHL) disease is a rare autosomal dominant syndrome that affects several organ systems and causes the formation of both benign and malignant tumors. The spectrum of VHL-associated tumors includes central nervous system (CNS) hemangioblastoma, retinal hemangioblastoma, pancreatic lesions including neuroendocrine tumors, pheochromocytomas, renal cell carcinomas (RCC), and endolymphatic sac tumors [1]. Approximately 7.6 % of patients with VHL disease may present with pancreatic symptoms during their initial visit. Cystic and/or solid pancreatic lesions are two potential symptoms of VHL disease. The majority of solid lesions are neuroendocrine tumors, while the majority of cystic lesions are simple cysts and serous cystadenomas. Here, we report a patient with VHL disease with a positive family history who presented with obstructive jaundice caused by multicystic pancreatic hamartoma. This case report was prepared following SCARE 2020 Guidelines and submitted after SCARE 2020 checks were completed [2].

## 2 Case presentation

A 39-year-old Indonesian male presented to the digestive clinic department with a 7-day history of jaundice. Jaundice was associated with epigastric discomfort, nausea, loss of appetite, and skin itch. He had a history of benign brain tumors and underwent craniotomies three times, at ages 16, 17, and 24. The type of brain tumor and specific procedure are not well known by the patient. Six months before the onset of jaundice, he was diagnosed with diabetes mellitus and began taking oral diabetic medications. The family history was remarkable; it was revealed that two of the four siblings of the patient have similar conditions. Both of his siblings underwent brain tumor resection, total pancreatectomy, and nephrectomy for Von Hippel-Lindau (VHL)-associated tumors. Vital signs were within normal range, and physical examination revealed jaundice on the skin and sclera, epigastric pain, and a palpable lump in the epigastric region and both of his flanks.

**Fig. 1:**
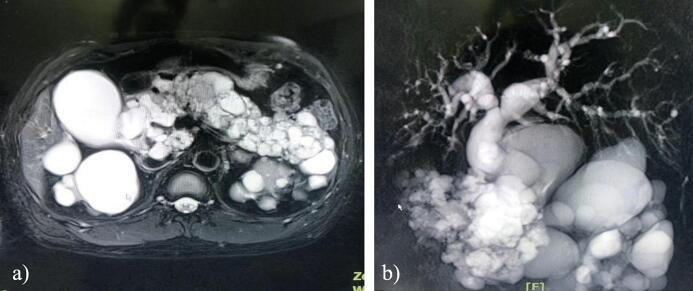
a) Contrast-Enhanced MRI Abdomen showed multiple cyst lesions in the pancreas and both kidneys, b) MRCP showed multiple pancreatic cysts with dilatation of the biliary tree.

**Fig. 2:**
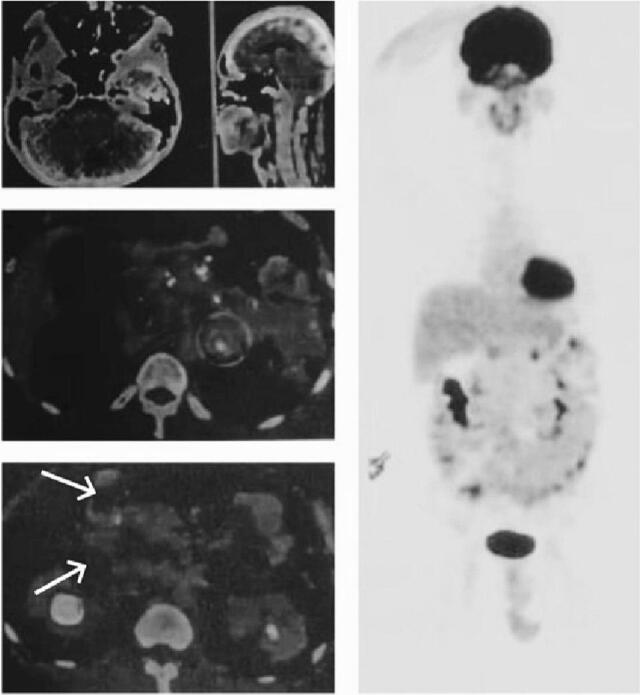
PET/CT scan demonstrated multiple hypodense nodules/cystic (white arrow) with multiple calcifications covering the entire pancreas.

Laboratory workup revealed elevated bilirubin levels, with serum total bilirubin of 6.54 mg/dL and conjugated bilirubin of 5.24 mg/dL. The contrast-enhanced MRI of abdomen with MRCP revealed dilatation of biliary tree consisting of right and left intrahepatic bile duct, common hepatic duct, cystic duct, and common bile duct without the presence of stone with suspicion partial obstruction by multiple cysts in the pancreas with diameters ranging from ±0.5–5 cm with ring enhancement, multiple cyst lesion with ring enhancement in both kidney and gallbladder hydrops with cholecystitis (Fig. 1). PET/CT scan showed an ametabolic hypodense lesion in the left side of the cerebellum, multiple hypodense nodules or cysts with multiple calcifications covering the entire pancreas, a solid hypermetabolic nodule in the left adrenal gland with suspicious pheochromocytomas, and an ametabolic hypodense multiple lesion in both kidneys. All radiologic findings correspond to von-Hippel-Lindau disease (Fig. 2).

Total pancreatoduodenectomy was decided after considering the lesion in the entire pancreas, which contains solid and cystic nodules, also the presence of endocrine pancreatic insufficiency. Following an evaluation of his fundamental condition and the exclusion of any contraindications, surgery was scheduled.

**Fig. 3:**
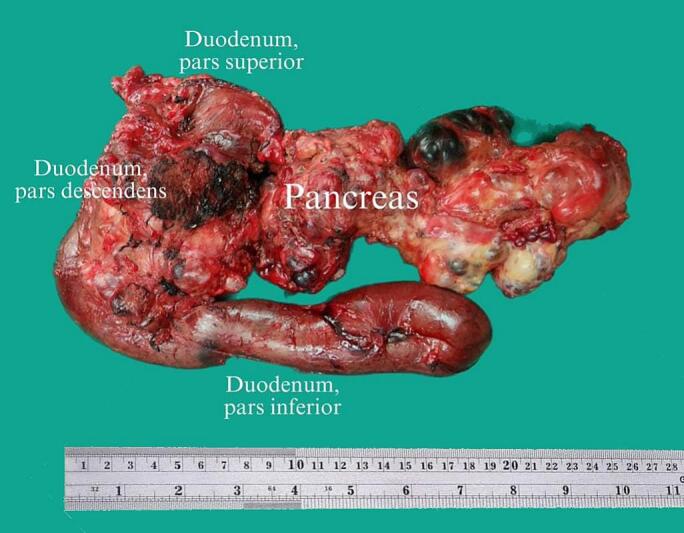
Gross findings of multiple cyst lesions in the pancreas.

**Fig. 4:**
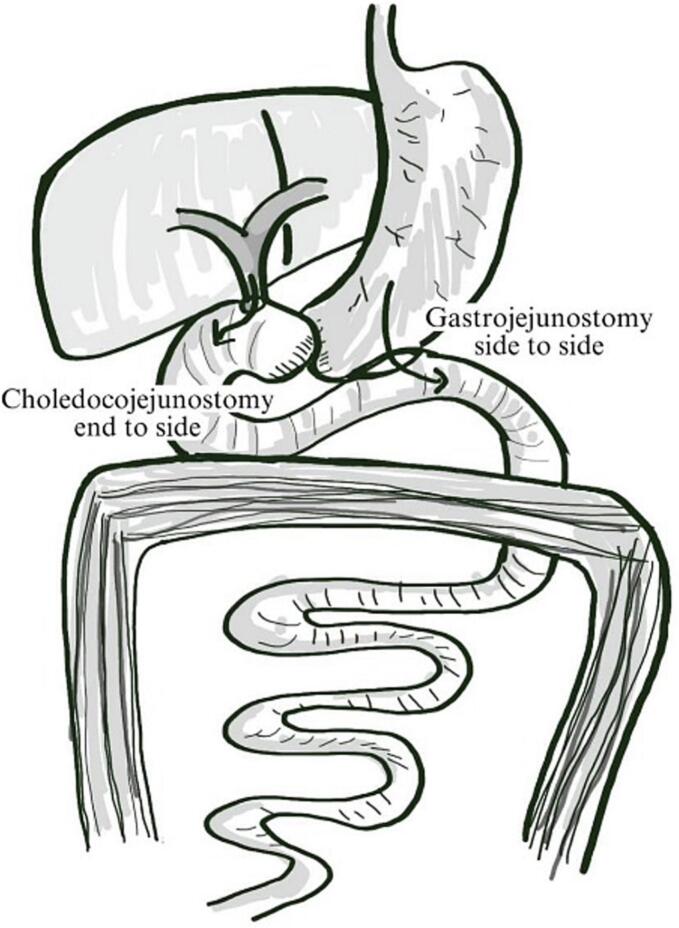
Illustration of total pancreaticoduodenectomy and reconstruction with end-to-side choledochojejunostomy, side-to-side gastrojejunostomy.

Intraoperatively, it was discovered that the cysts were seen in the entire pancreas with dilatation of biliary tree (Fig. 3). Therefore, total pancreaticoduodenectomy was performed and followed by reconstruction with end-to-side choledochojejunostomy, side-to-side gastrojejunostomy, and subhepatic drainage was placed (Fig. 4).

**Fig. 5:**
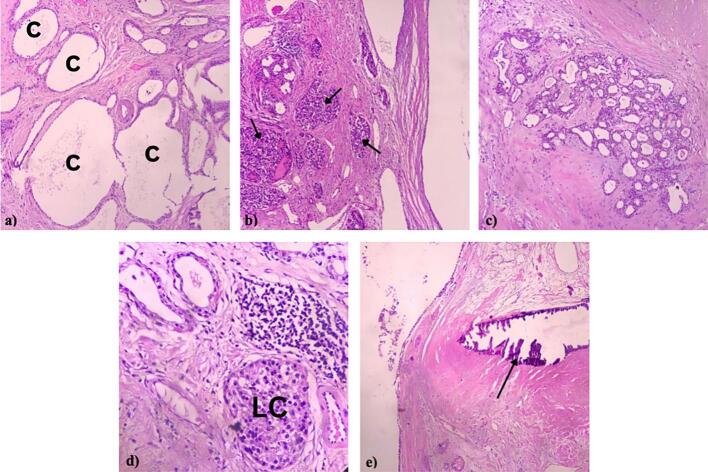
Microscopic findings of multicystic pancreatic hamartoma with neuroendocrine cell hyperplasia; a) Multiple serous cystic (C), b) neuroendocrine cell hyperplasia (arrowhead), c) pancreatic hamartoma, d) lesion still showed islet of Langerhans (LC), e) pancreatic calcifications (arrowhead).

The histopathological findings showed multiple serous cystic neoplasms with calcification. Immunohistochemically, the report further confirmed multicystic pancreatic hamartoma with neuroendocrine cell hyperplasia and the lesion still showed an islet of Langerhans structure. Diagnosis is supported by diffuse positive in Cytokeratin 19 (CK19) and CK7 in cystic lesions, negative CEA, positive synaptophysin and chromogranin in nodular lesions, and positive Ki67% (0.2 %) which exclude adenocarcinoma or neuroendocrine tumor findings (Fig. 5).

The management of the patient is optimally performed by an interprofessional healthcare team. Glycemic control after total pancreatectomy is handled and monitored by an endocrinologist. The insulin regimen prescribed for the patient was as follows: 10 IU of basal insulin taken in the evening, 8 IU and 10 IU of rapid-acting insulin given bolus during breakfast and dinner, respectively. The treatment for exocrine insufficiency was enzyme-substitution drug, which contains amylase, protease, deoxycholic acid, dimethylpolysiloxane, vitamin B1, vitamin B2, vitamin B6, niacinamide, and calcium pantothenate. In addition, with radiologic findings of multiple cyst lesions in both kidneys and a solid nodule in the left adrenal gland with suspicion of pheochromocytoma, close monitoring by a urologic surgeon was advised.

After three months of follow-up, the patient's main complaints were unstable blood glucose levels with variations of about 60 up to 400 mg/dL and an average of 200 mg/dL checked by a home-use blood glucose meter. Moreover, the patient report having at least three loose bowel movements each day and oftentimes fatty stool (steatorrhea) after overindulging in a high-fat diet. At six months follow-up, the blood glucose was controlled within the range 80-200 mg/dL and could take a normal low-fat diet without steatorrhea.

## 3 Clinical discussion

Von Hippel-Lindau (VHL) disease can be known as a rare inherited autosomal dominant syndrome that affects several organ systems and causes the formation of both benign and malignant tumors. The alteration is caused by VHL tumor suppressor gene loss on the short arm of chromosome 3, which lead to hemangioblastomas of the central nervous system, retinal hemangioblastomas, renal cell carcinomas (RCC), pancreatic cysts and tumors, pheochromocytoma, and endolymphatic sac tumors [3]. These kind of hemangioblastomas can be found in the cerebellum (16–69 %), brainstem (5–22 %), cauda equine (11 %), spinal cord (13–53 %), or supratentorial area (1–7 %). As evidenced by the patient, the cerebellum and spinal cord are the most typical locations for hemangioblastoma growth. Hemangioblastomas are most commonly identified in the temporal peripheral retina, however up to 15 % of individuals may exhibit the condition in the justapapillary area [4].

However, there are no structural defects on either retina of our patient, but he had a history of benign brain tumors and had craniotomies three times at the ages of 16, 17, and 24. The type of brain tumor and the specific procedure is not well-known by the patient. Hemangioblastomas of the CNS typically appear between the ages of 10 and 30 during childhood or early adolescence. Symptoms of hemangioblastomas are caused by the formation of tumors in the intracranial space and spinal cord, whereas this patient didn't exhibit spinal cord lesion symptoms [5].

The clinical diagnostic criteria for VHL disease, as defined by Melmon and Rosen in 1964, are explained as (i) greater than one CNS hemangioblastoma; (ii) CNS hemangioblastoma in combination with visceral manifestations of VHL disease; and (iii) any manifestation and a known family history of VHL disease [6]. This patient had two of four siblings with similar conditions.

Renal cell carcinoma, the primary malignant tumor in von Hippel-Lindau disease, frequently displays the highest fatality rates. Between 24 and 45 % of the population may have it. The overall prevalence of renal lesions, including renal cysts, is 60 %, and the average age at presentation is 39 years. Bilateral, multifocal, and multiple lesions of the kidney are common. They mostly display no symptoms for significant periods and only become noticeable in late instances. The treatment suggestions vary on the size of the tumor. For carcinomas with a maximum diameter of 3 cm, nephron-sparing surgery is suggested. Nephron-sparing or renal-function-preserving resection is intended to limit the risk of metastases while preserving kidney function. Large carcinomas (>3 cm), particularly when multifocal involvement is confirmed, carry a higher risk of metastasis. In extremely rare instances where the kidney cannot be saved, a complete nephrectomy is the sole treatment option [7]. In this case, with radiologic findings of multiple cyst lesions in both kidneys and a solid nodule in the left adrenal gland with suspicion of pheochromocytoma, close observation by a urologic surgeon was advised.

Approximately 7.6 % of patients with VHL disease may present just with pancreatic symptoms during their initial visit. Multiple pancreatic cysts without prior pancreatic inflammatory episodes should suggest VHL disease and prompt a genetic test, according to clinical presentation. As soon as the diagnosis is made, all potential family members must be screened, and those who are affected must receive genetic counseling and strict follow-up care in order to treat the disease's potentially fatal CNS and visceral manifestations. [8]

Pancreatic lesions include simple unilocular cystic lesions or simple pancreatic cysts, serous or mucinous cystadenomas, neuroendocrine tumors (NET), hemangioblastomas, renal cell cancer metastases, and adenocarcinoma. Cystic and/or solid pancreatic lesions are two potential symptoms of VHL disease. Combination lesions are recorded in 11.5 % of VHL disease patients. The majority of solid lesions are neuroendocrine tumors, while the majority of cystic lesions are simple cysts and serous cystadenomas [9]. Based on the potential for pancreatic malignancy, including pancreatic cancer, mucinous cystadenocarcinoma, or mucinous cystadenoma with malignant potential, it is recommended to consider resection of pancreatic tumors that lack a definitive diagnosis of serous cystic neoplasms [10]. This approach can promote optimal management and decrease the risk of tumor progression or malignant transformation^.^ This patient was diagnosed with multicystic pancreatic hamartoma and neuroendocrine cell hyperplasia based on histopathology and immunochemistry, which exclude malignant lesions. A hamartoma is a benign tumor-like formation composed of a heterogeneous collection of cells and tissues that are regularly present in the area of the body where the growth forms. Hamartomas are mostly asymptomatic and rarely cause complications. Nevertheless, certain lesions can develop to massive proportions, causing internal disfigurement and organ compression [11,12]. Through this process, it was discovered that this patient had obstructive jaundice that caused an obstruction of the common bile duct by pancreas hamartoma.

The pancreas is known to be commonly differentiated into exocrine portion (acinar and duct tissue) and endocrine portion (islets of Langerhans). The immunohistochemistry of CK-19 and CK-7, two typical epithelial markers, are often expressed in the exocrine ducts of the pancreas but not in the exocrine acinar and endocrine islet cells [14]. Carcinoembryonic antigen (CEA) is usually considered an epithelial marker with expression in various adenocarcinomas [13]. Chromogranin A (CgA) and synaptophysin are currently considered to be the most sensitive and specific markers for neuroendocrine portion. Furthermore, Neuron-Specific Enolase (NSE) is a highly specific marker for neurons and peripheral neuroendocrine cells. As a result of the findings of NSE in certain tissues under normal settings, elevated bodily fluid levels of NSE may occur with malignant growth and may thus be useful for the diagnosis, staging, and management of associated neuroendocrine tumors (NET) [15]. Grading NETs are based on mitotic count and the Ki-67 index. Ki-67 is a nuclear protein expressed in proliferating cells. The Ki-67 index stratifies PanNET into low-grade tumors (Ki-67 < 3 %), intermediate-grade tumors (Ki67 3–20 %), and high-grade carcinoma (Ki-67 > 20 %) [16]. Therefore in this patient, negative staining for CEA excludes the likelihood of adenocarcinoma and low Ki-67 index (0,2 %) with positive Chromogranin A and Synaptophysin indicating neuroendocrine cell hyperplasia.

In VHL disease, the majority of pancreatic lesions are asymptomatic. When symptomatic, they frequently exhibit obstructive jaundice, epigastric pain, diarrhea, dyspepsia, and other nebulous symptoms, as presented in this patient. In VHL disease, common bile duct obstruction caused by a serous cystadenoma can result in obstructive jaundice. They infrequently develop symptoms related to endocrine/exocrine pancreatic insufficiency or other organ compression, leading to pancreatitis, abdominal pain, and intestinal sub-occlusion. Enzyme replacement is used to treat exocrine pancreatic insufficiency. Even when the parenchyma is replaced completely by cysts, the chance of developing diabetes is minimal. If the cystic lesion compresses adjacent tissues and the patient develops painful or obstructive symptoms, excision of the cystic lesion is recommended [17].

Imaging is essential for diagnosing and differentiating benign lesions or malignant tumors, as well as detecting any change in the basic features of lesions. The optimal identification of pancreatic lesions was achieved by systematically examining individuals with VHL illness using CT scans by trained radiologists. CT and MRI have been used to characterize pancreatic lesions. MRI is the preferred imaging technique due to the absence of radiation and the current literature guideline for annual abdominal monitoring. Characteristic MRI findings can differ depending on the lesion [18]. The patient's PET/CT scan revealed an ametabolic hypodense lesion on the left side of the cerebellum, multiple hypodense nodules or cysts with multiple calcifications covering the entire pancreas, a hypermetabolic solid nodule in the left adrenal gland with suspicious of pheochromocytoma, and an ametabolic hypodense multiple lesions in both kidneys. All radiologic findings correspond to VHL disease.

The surgical approach for pancreatic cysts is tailored based on their location and extent. A distal pancreatectomy is performed when the cyst is located in the body of the pancreas. A median pancreatectomy or enucleation may be considered for small lesions in the body and neck of the pancreas. Following this procedure, the proximal pancreatic segment is closed, and the distal segment is drained with a Roux-en-Y pancreatojejunostomy. Pancreatoduodenectomy (PD) is the preferred approach for tumors located in the head and/or uncinate process. This procedure is performed with or without pylorus preservation, and either pancreaticogastrostomy (PG) or pancreatojejunostomy (PJ) based on surgeon preference. There was difficult to find smooth tissue of pancreas to do the anastomosis of pancreas if the lesion covered the whole pancreas. In this case, the cysts were seen in the entire pancreas with dilatation of the biliary tree, with only a narrow gap left in hepatoduodenal space. The surgical options were to release the symptoms by biliary bypasses such as Roux en Y hepaticojejunostomy (RYHJ) or total pancreatoduodenectomy. The RYHJ procedure was difficult because of limited space in the hepatoduodenal area due to massive enlargement of the pancreas. Total pancreatectomy was performed, because of two reasons. First after considering the lesion that could progress to neuroendocrine carcinoma or mucinous cystadenocarcinoma. The second reason was the endocrine function of pancreas was also deteriorating in the last six months, because of increasing blood glucose and became diabetes mellitus [19].

In the long-term follow-up post-total pancreatectomy, insulin-dependent diabetes mellitus (IDDM) with unstable and difficult-to-control blood glucose levels made significant contributions to morbidity and mortality. But nowadays, the medical care of total pancreatectomy has improved as a result of advancements in surgical procedures and glycemic monitoring, as well as the availability of synthetic insulin and pancreatic enzymes [18]. The main complaints of this patient were unstable and difficult-to-control blood glucose levels with variations of about 60 up to 400 mg/dL and an average of 200 mg/dL checked by a home-use blood glucose meter. Glycemic control had achieved after six months of taking insulin regularly.

Pancreatic diabetes mellitus is characterized by a total absence of endogenous insulin and glucagon, resulting in frequent and severe hypoglycemia and difficult-to-control hyperglycemia (brittle diabetes) [21,22]. Recent developments in long-acting insulin formulations and the enhancement of pancreatic enzyme supplements have provided total pancreatectomy patients with safety without the occurrence of significant fluctuations and intestinal malabsorption issues. The median amount of total insulin units reported in prior investigations varied between 25 and 34 IU, whereas the median number of long-acting insulin units varied between 7 and 12 IU. In earlier research, the median number of rapid-acting insulin units varied between 18 and 21 IU. Due to a rise in the expression of peripheral insulin receptors, the need for insulin was often less than expected following total pancreatectomy. According to earlier research, the need for insulin was often less than expected following total pancreatectomy due to a rise in the expression of peripheral insulin receptors and people with pancreatic diabetes required less insulin per day than those with type 1 DM [21]. The insulin regimen prescribed for the patient was 10 IU of basal insulin and 18 IU rapid-acting insulin.

The cause of diarrhea following total pancreatectomy was exocrine insufficiency and less often a possibility of nerve injury surrounding the superior mesenteric artery (SMA) which result in decreased bowel control. The treatment of exocrine insufficiency required a high dosage of pancreatic enzymes to prevent weight loss and minimize diarrhea. Exocrine replacement with daily pancreatic enzyme supplementation (80,000–150,000 IU) was necessary. Median weight loss in post-total pancreatectomy patients was 5–15 kg. A daily schedule of many calorie-dense meals might minimize nutritional deficits and weight loss [ 19,20].

## 4 Conclusion

In conclusion, we have documented a rare case of von Hippel-Lindau disease in which multiple pancreatic cysts and multiple renal cysts were identified. The pancreatic lesions observed throughout the whole pancreas were responsible for the biliary tree dilatation. Total pancreatoduodenectomy could be performed to relief the symptom severity and avoid possibility of malignant changes.

## CRediT authorship contribution statement

Wifanto Saditya Jeo, Khalikul Razi, and Andre Setiawan performed the surgery.

Wifanto Saditya Jeo, Khalikul Razi, Andre Setiawan, Natasya, and Welly Hartono Ruslim performed the conception, design, drafting of the article, and critical revision for important intellectual content.

All authors read and approved the final manuscript.

## Informed consent

Written informed consent was obtained from the patient's parents/legal guardian for the publication of this case report and accompanying images. A copy of the written consent is available for review by the Editor-in-Chief of this journal on request.

## Ethical approval

No ethical approval was necessary for the treatment or investigation of this patient.

## Source of funding

This study is self-funded and received no external financial support from the public or commercial sectors.

## Research registration

N/A

## Guarantor

Wifanto Saditya Jeo is a guarantor of this study.

## Declaration of competing interest

There is no conflict of interest associated with this publication.
